# Effect of Lipopolysaccharide on Glucocorticoid Receptor Function in Control Nasal Mucosa Fibroblasts and in Fibroblasts from Patients with Chronic Rhinosinusitis with Nasal Polyps and Asthma

**DOI:** 10.1371/journal.pone.0125443

**Published:** 2015-05-05

**Authors:** Laura Fernández-Bertolín, Joaquim Mullol, Mireya Fuentes-Prado, Jordi Roca-Ferrer, Isam Alobid, César Picado, Laura Pujols

**Affiliations:** 1 Clinical and Experimental Respiratory Immunoallergy, Centre de Recerca Biomèdica CELLEX, Institut d’Investigacions Biomèdiques August Pi i Sunyer (IDIBAPS), Barcelona, Spain; 2 Centro de Investigaciones Biomédicas en Red de Enfermedades Respiratorias (CIBERes), Barcelona, Spain; 3 Rhinology Unit and Smell Clinic, ENT Department, Hospital Clínic, Barcelona, Spain; 4 Allergy Unit, Pneumology and Allergy Department, Hospital Clínic, Universitat de Barcelona, Barcelona, Spain; Baylor College of Medicine, UNITED STATES

## Abstract

**Background:**

Chronic rhinosinusitis with nasal polyps (CRSwNP) is a chronic inflammatory disease of the upper airways frequently associated with asthma. Bacterial infection is a feature of CRSwNP that can aggravate the disease and the response to glucocorticoid treatment.

**Objective:**

We examined whether the bacterial product lipopolysaccharide (LPS) reduces glucocorticoid receptor (GR) function in control nasal mucosa (NM) fibroblasts and in nasal polyp (NP) fibroblasts from patients with CRSwNP and asthma.

**Methods:**

NP (n = 12) and NM fibroblasts (n = 10) were *in vitro* pre-incubated with LPS (24 hours) prior to the addition of dexamethasone. Cytokine/chemokine secretion was measured by ELISA and Cytometric Bead Array. GRα, GRβ, mitogen-activated protein-kinase phosphatase-1 (MKP-1) and glucocorticoid-induced leucine zipper (GILZ) expression was measured by RT-PCR and immunoblotting, GRα nuclear translocation by immunocytochemistry, and GRβ localization by immunoblotting. The role of MKP-1 and GILZ on dexamethasone-mediated cytokine inhibition was analyzed by small interfering RNA silencing.

**Results:**

Pre-incubation of nasal fibroblasts with LPS enhanced the secretion of IL-6, CXCL8, RANTES, and GM-CSF induced by FBS. FBS-induced CXCL8 secretion was higher in NP than in NM fibroblasts. LPS effects on IL-6 and CXCL8 were mediated via activation of p38α/β MAPK and IKK/NF-κB pathways. Additionally, LPS pre-incubation: 1) reduced dexamethasone’s capacity to inhibit FBS-induced IL-6, CXCL8 and RANTES, 2) reduced dexamethasone-induced GRα nuclear translocation (only in NM fibroblasts), 3) did not alter GRα/GRβ expression, 4) decreased GILZ expression, and 5) did not affect dexamethasone’s capacity to induce MKP-1 and GILZ expression. MKP-1 knockdown reduced dexamethasone’s capacity to suppress FBS-induced CXCL8 release.

**Conclusion:**

The bacterial product LPS negatively affects GR function in control NM and NP fibroblasts by interfering with the capacity of the activated receptor to inhibit the production of pro-inflammatory mediators. This study contributes to the understanding of how bacterial infection of the upper airways may limit the efficacy of glucocorticoid treatment.

## Introduction

Chronic rhinosinusitis with nasal polyps (CRSwNP) is a chronic inflammatory disease of the sinus mucosa frequently associated with asthma and with aspirin-exacerbated respiratory disease [1]. Asthma and aspirin-exacerbated respiratory disease co-morbidities are a link for CRSwNP severity [2]. In addition to persistent mucosal inflammation, microbial infection by both Gram-positive and Gram-negative bacteria is a feature of both CRSwNP and chronic rhinosinusitis without nasal polyps [3–9]. There is emerging evidence that microorganisms play an important role in the exacerbation and perpetuation of mucosal inflammation. Intranasal glucocorticoids, with/without administration of short courses of oral glucocorticoids, are the first-line treatment for CRSwNP [1,10]. However, some patients with CRSwNP are not adequately controlled despite guideline-based treatment with glucocorticoids. Viral and bacterial infections, and exposure of the airways to endotoxins contribute to glucocorticoid insensitivity [11–14].

Glucocorticoids exert their effects by binding to a cytoplasmic receptor, namely the glucocorticoid receptor (GR) α. The glucocorticoid-bound GRα rapidly translocates into the nucleus and modulates, either positively or negatively, the expression of target genes. Glucocorticoid anti-inflammatory effects are explained by inhibition of proinflammatory gene expression through blockade of proinflammatory transcription factors, such as activating protein-1 and nuclear factor-κB (NF-κB). Glucocorticoid anti-inflammatory effects are also explained by transcriptional activation (transactivation) of anti-inflammatory genes [15], such as the *mitogen-activated protein kinase (MAPK) phosphatase-1 (MKP-1)* and the *glucocorticoid-induced leucine zipper (GILZ)* [16,17].

The GR is a target for infectious agents. Bacterial microorganisms and their breakdown products such as lipopolysaccharide (LPS), a cell wall component of Gram-negative bacteria, decrease GR ligand affinity and GR number and affinity [18]. Both LPS and the Gram-negative bacterium *Haemophilus parainfluenzae* attenuate *MKP-1* induction by dexamethasone in bronchoalveolar lavage (BAL) macrophages from asthmatic patients [13], and respiratory viruses reduce *MKP-1* [11,19] and *GILZ* [19] induction by dexamethasone in bronchial epithelial cells.

Human nasal fibroblasts release a variety of proinflammatory and profibrotic mediators that can contribute to upper airways inflammation and remodeling [20,21]. Nasal fibroblasts respond to LPS via recognition of Toll-like receptors by producing inflammatory mediators such as the chemoattractants MCP-4, eotaxin and regulated on activation normal T cell expressed and secreted (RANTES), IL-6 and CXCL8, and growth factors such as the granulocyte/macrophage colony-stimulating factor (GM-CSF) [22–24]. We have previously reported that nasal polyp (NP) fibroblasts from patients with CRSwNP and asthma have a lower sensitivity to glucocorticoids, compared to nasal mucosa (NM) fibroblasts from control patients [21,25].

We hypothesized that exposure of nasal fibroblasts to LPS reduces GR anti-inflammatory functions, and that the effects of LPS on GR function are modulated by the presence of a pre-existing inflammatory process, such as that of patients with CRSwNP and asthma. Therefore, the objective of this study was to examine the effects of LPS on GR function in *in vitro* cultured control NM fibroblasts and in NP fibroblasts from patients with CRSwNP and asthma. Specifically, we determined the effect of LPS on glucocorticoid-mediated inhibition of proinflammatory cytokines, as well as on GR expression, nuclear translocation and transactivation of anti-inflammatory genes. We finally evaluated the role of GR transactivation of MKP-1 and GILZ on the inhibition of proinflammatory cytokine release mediated by glucocorticoids.

## Methods

### Reagents

Dulbecco’s modified Eagle’s medium (DMEM) was obtained from Lonza (Verviers, Belgium), fetal bovine serum (FBS) from Biological Industries (Beit Haemek, Israel). Charcoal-stripped (steroid-free) FBS (csFBS), trypsin-EDTA, penicillin, streptomycin, HEPES, RT-PCR and immunofluorescence and Western Blot reagents, small interfering RNAs (siRNA), and all other transfection reagents were purchased from Life Technologies (Paisley, UK). Dexamethasone (Fortecortin) was obtained from Merck (Darmstadt, Germany) and amphotericin B and LPS (from *Escherichia coli* 0111:B4) from Sigma-Aldrich (St Louis, Missouri, USA). The RNeasy Mini kit was purchased from Qiagen Inc. (Valencia, California, USA). All antibodies were from Santa Cruz Biotechnology (Santa Cruz, California, USA), unless otherwise specified. RANTES, eotaxin, GM-CSF, and CXCL8 BD Cytometric Bead Array Flex Sets were from BD Biosciences (San Diego, California, USA), and IL-6 and CXCL8 DuoSet commercial enzyme-linked immunosorbent assay (ELISA) kits were from R&D Systems (Abingdon, UK).

### Subjects

Human NP tissue was obtained from 12 subjects with CRSwNP and asthma undergoing functional endoscopic sinus surgery (S1 Table). Patients were selected on the basis of a medical history consistent with severe CRSwNP, based on established criteria described elsewhere [1,21]. The diagnosis of asthma was established on the basis of the clinical history and the demonstration of a reversible bronchial obstruction. Diagnosis of aspirin-exacerbated respiratory disease was made on the basis of a clear-cut history of asthma attacks precipitated by non-steroidal anti-inflammatory drugs (NSAIDs), and confirmed by aspirin nasal challenge in patients with an isolated episode of NSAID-induced asthma exacerbation [26]. The histology of the NPs of CRSwNP patients is predominantly eosinophilic [27,28]. Control NM was obtained from the inferior turbinate of 10 subjects undergoing turbinectomy for turbinate hypertrophy with or without septoplasty for nasal septum deviation. All patients gave written informed consent to participate in the study, which was approved by the Ethics Committee of Hospital Clínic, Barcelona (approval number: 2008/4291).

### Fibroblast culture and treatments

Nasal fibroblasts from the tissue fragments were isolated using an explant outgrowth method and experiments were carried out between passages 4 to 8. Cell specificity was confirmed by positive vimentin and negative cytokeratin staining, as previously described [21]. At sub-confluence, fibroblasts were pre-incubated with 10% csFBS-supplemented medium or 10% FBS-supplemented medium (for MKP-1 protein analysis) [25] in the absence or presence of LPS (10 μg/ml) for 24 hours. Then, dexamethasone was added and GR nuclear translocation and transactivation was analyzed. For cytokine determination studies, after pre-incubation with/without LPS, the cell medium was removed and changed to either 10% FBS-supplemented medium or LPS (in 10% csFBS) in the absence or presence of dexamethasone for 24 hours. In some experiments, cells were pre-incubated for 1 hour with the p38α/β MAPK inhibitor SB203580 (10 μM), the c-Jun N-terminal kinase (JNK) inhibitor SP600125 (20 μM) or the IκB kinase (IKK)/NF-κB inhibitor BMS-345541 (2 μM) before LPS incubation.

### Small interfering RNA silencing

Thirty to 50% confluent nasal fibroblasts were transfected with 20 nM of either MKP-1 or GILZ Silencer Select pre-designed siRNA or Silencer Select negative control siRNA using lipofectamine RNAiMAX, as previously reported [29]. Twenty-four hours after transfection, cells were pre-incubated in the absence or presence of LPS (10 μg/ml) for 24 hours. Forty-eight hours after transfection, cells were incubated with 10% FBS-supplemented medium with/without dexamethasone (10^–6^ M) for one (for MKP-1 protein), six (for GILZ protein) or twenty-four (for CXCL8 release) hours.

### Cell viability

Cell viability after treatment was determined using the colorimetric Cell Proliferation Kit II (XTT, Roche Diagnostics GmbH, Mannheim, Germany), as previously reported [20].

### Cytokine determination

RANTES, eotaxin, GM-CSF and CXCL8 were quantified using a specific BD Cytometric Bead Array (CBA) Flex Set (BD Biosciences, San Diego, California, USA), as previously reported [20]. IL-6 and CXCL8 levels were measured by using commercial enzyme-linked immunosorbent assay (ELISA) DuoSet kits (R&D Systems, Abingdon, UK), according to the manufacturer’s instructions. Cytokine production was corrected by cell number as determined by the XTT assay.

### Quantitative real-time RT-PCR

Total RNA was isolated and reverse transcribed to cDNA [29]. *GRα*, *GRβ*, *MKP-1*, *GILZ*, and *RNA polymerase II (RPII)* mRNA expression was analyzed by real-time PCR using TaqMan Fast Universal PCR Master Mix and either customized (for *GRα*) or predesigned (for all other genes) TaqMan Gene Expression Assays (Life Technologies) [25]. Target gene expression was normalized to *RPII*, as previously reported [25,29].

### Western Blot analysis

Whole cell protein lysates and nuclear and cytoplasmic cellular extracts from nasal fibroblasts were obtained following previously reported protocols [25,29]. Nitrocellulose membranes were incubated with primary antibodies against MKP-1 (C-19), GILZ (G-5), GR (E-20), GRβ (Pierce Biotechnology, Rockford, IL, USA), β-actin (AC-15, Sigma-Aldrich), lamin-B (M-20), and α-tubulin (B-7). Protein signals were normalized to those of α-tubulin, β-actin, or lamin-B, as appropriate.

### Immunofluorescence

Cells were incubated with an antibody against GR (E-20) and a secondary antibody conjugated to Alexa 488, as previously reported [25]. The mean fluorescence intensity of Alexa 488 staining was assessed by analysis software within the nuclear regions of the cells. Forty to 60 cells were analyzed for each patient and condition studied and expressed as mean fluorescence intensity per mean area relative to non-treated cells at baseline (0 hours). The values of GR nuclear translocation induced by dexamethasone over time were also divided by the respective (medium or LPS) baseline values.

### Statistical data analysis

Data are given as median and interquartile range. Multiple comparisons were analyzed by using one-way analysis of variance with Tukey/Newman-Keuls post hoc analysis or with Kruskal-Wallis with Dunn’s post hoc analysis, as appropriate. Comparisons between two groups were made with the *t* test or the Mann-Whitney U test, as appropriate. Statistical analyses were performed with GraphPad Prism software (GraphPad Software, La Jolla, Calif; http://www.graphpad.com). Statistical significance was set at *P*<.05.

## Results

### Effect of LPS on cytokine secretion

**Fig 1:**
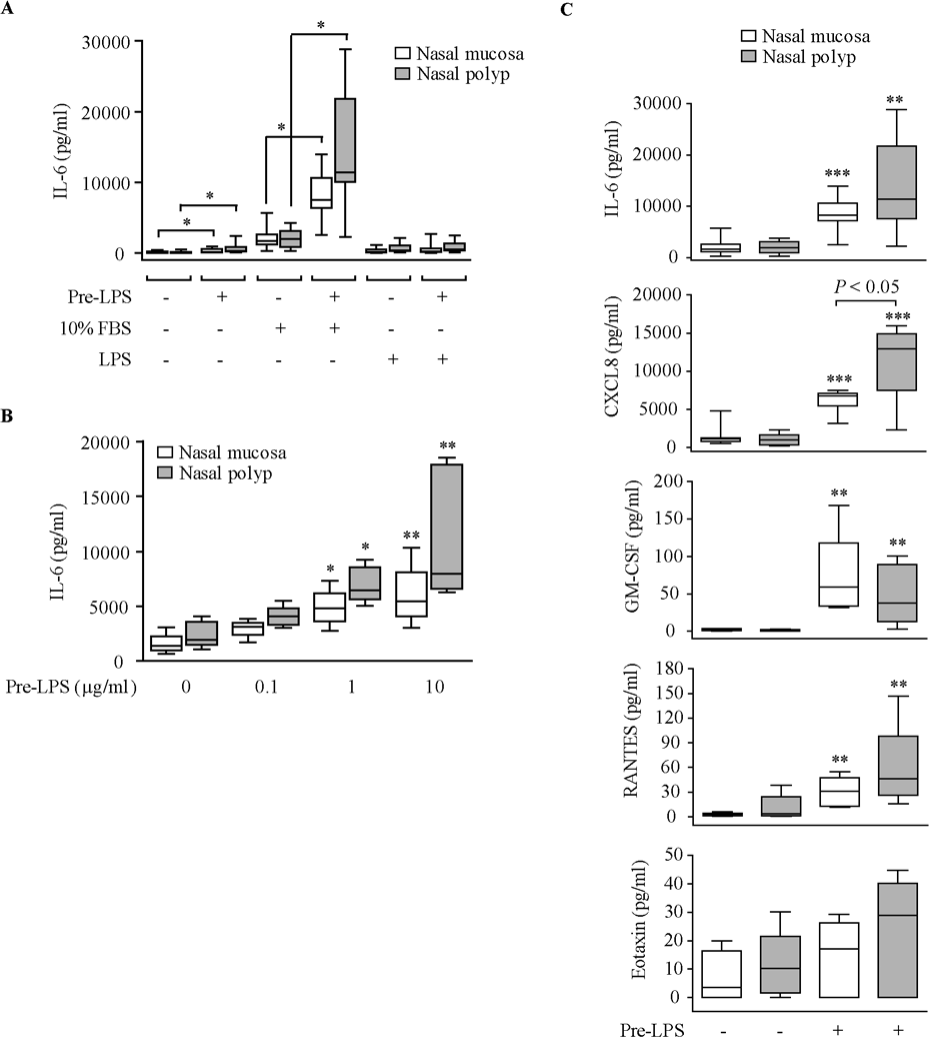
Effect of LPS on cytokine production. (A) ELISA quantification of IL-6 production in cell supernatants of NM and NP fibroblasts (n = 8 each) pre-incubated with 10% csFBS-supplemented medium with/without 10 μg/ml LPS (Pre-LPS, 24 hours) and then incubated with LPS or 10% FBS-supplemented medium (10% FBS) for 24 hours. *P<.05. (B) Effect of increasing LPS concentrations on 10% FBS-induced IL-6 production (NM and NP, n = 4–5). (C) Effect of LPS (10 μg/ml) on 10% FBS-induced cytokine/chemokine production (NM and NP, n = 5–8). *P<.05, ** *P*<.01, and ***P<.001 *versus* medium-treated cells.

We first investigated the effect of LPS on the production of IL-6 by nasal fibroblasts. Maximal IL-6 production was found in cells pre-incubated with LPS and then stimulated with 10% FBS (Fig 1A). Pre-incubation of cells with increasing LPS concentrations resulted in higher induction of IL-6 secretion by 10% FBS (Fig 1B). LPS did not induce cytotoxicity in nasal fibroblasts as determined by the XTT metabolic assay (data not shown). LPS pre-incubation also enhanced CXCL8, RANTES and GM-CSF secretion by 10% FBS, but did not increase eotaxin release (Fig 1C). In cells pre-incubated with LPS, NP fibroblasts secreted higher levels of CXCL8 in response to 10% FBS than the control NM fibroblasts.

**Fig 2:**
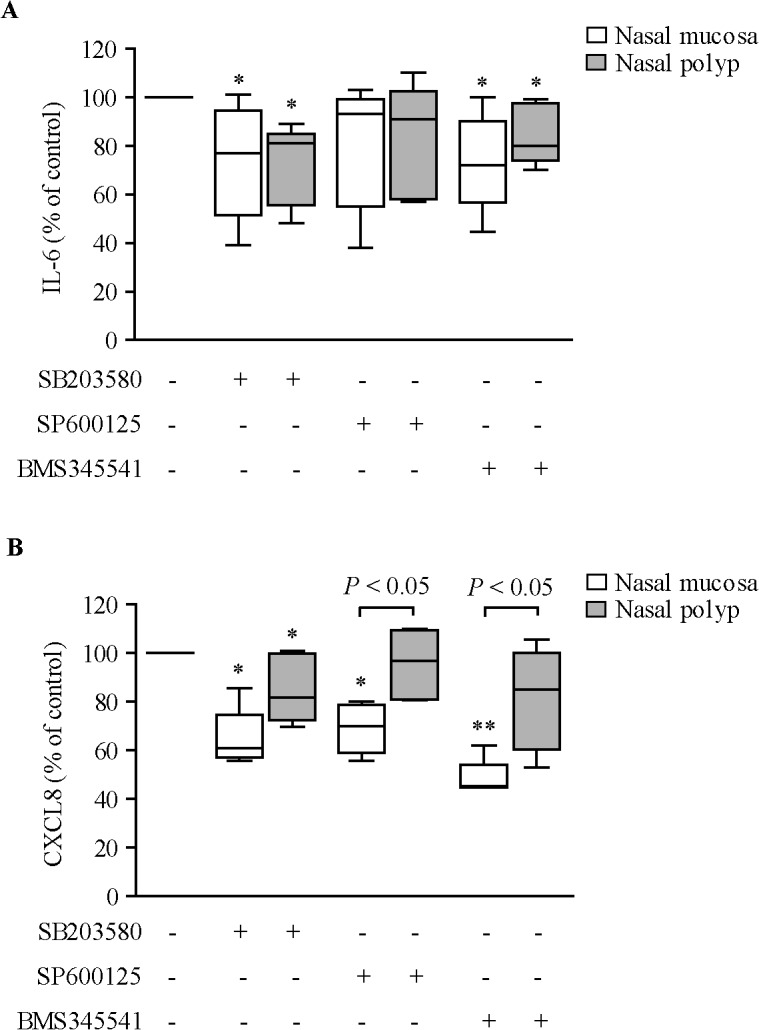
Effect of kinase inhibitors on LPS-induced IL-6 and CXCL8 secretion. ELISA quantification of IL-6 (A) and CXCL8 (B) production in cell supernatants of NM and NP fibroblasts (n = 4–6) incubated with 10% csFBS-supplemented medium with/without 10 μM SB203580 (p38α/β MAPK inhibitor), 20 μM SP600125 (JNK inhibitor) or 2 μM BMS-345541 (IκB kinase/NF-κB inhibitor) for 1 hour prior to LPS (10 μg/ml) addition for 24 hours. *P<.05 and **P<.01 *versus* LPS alone (100%).

We examined the intracellular signaling pathways involved in LPS effects. P38α/β MAPK (SB203580) and IKK/NF-κB (BMS-34554) but not JNK (SP600125) inhibitors partially suppressed the induction of IL-6 secretion by LPS in both NM and NP fibroblasts (Fig 2A). SB203580 partially suppressed CXCL8 secretion in both NM and NP fibroblasts (Fig 2B). However, BMS-34554 and SP600125 suppressed CXCL8 only in NM fibroblasts.

### Effect of LPS on dexamethasone inhibition of cytokine secretion

**Fig 3:**
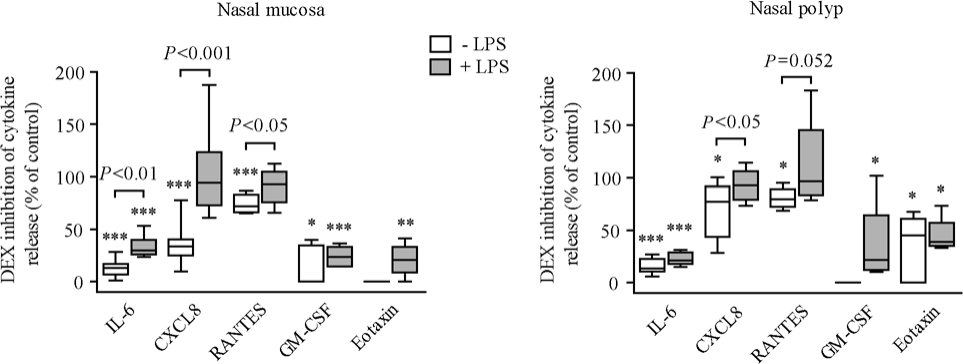
Effect of LPS on dexamethasone inhibition of cytokine secretion. ELISA quantification of cytokine/chemokine production in cell supernatants of NM and NP fibroblasts (n = 4–8) pre-incubated with 10% csFBS-supplemented medium with/without LPS (10 μg/ml, 24 hours) and then incubated with 10% FBS-supplemented medium with/without dexamethasone (DEX: 10^–6^ M) for 24 hours. Cytokine production normalized to each respective control. *P<.05, **P<.01, and ***P<.001 *versus* medium (no DEX).

Dexamethasone concentration-dependently inhibited the induction of IL-6 production by 10% FBS in both medium and LPS-pre-treated NM and NP fibroblasts (S1 Fig). LPS pre-incubation significantly attenuated dexamethasone inhibition of FBS-induced IL-6 secretion in NM but not in NP fibroblasts. In the absence of LPS pre-treatment, dexamethasone inhibited FBS-induced CXCL8, RANTES, GM-CSF and eotaxin secretion (Fig 3). CXCL8 was less inhibited by dexamethasone in NP [22.69% (median) inhibition; *P*<.05] than in NM (66.57% inhibition) fibroblasts. LPS pre-incubation abrogated dexamethasone inhibition of CXCL8 secretion in both NM and NP fibroblasts, and attenuated dexamethasone inhibition of RANTES in NM fibroblasts (Fig 3).

### Effect of LPS on GR expression and nuclear translocation

Baseline *GRα* and *GRβ* mRNA and protein levels did not differ between NM and NP fibroblasts (data not shown). Moreover, LPS pre-incubation did not affect *GRα* and *GRβ* mRNA and protein levels (S2 Fig).

**Fig 4:**
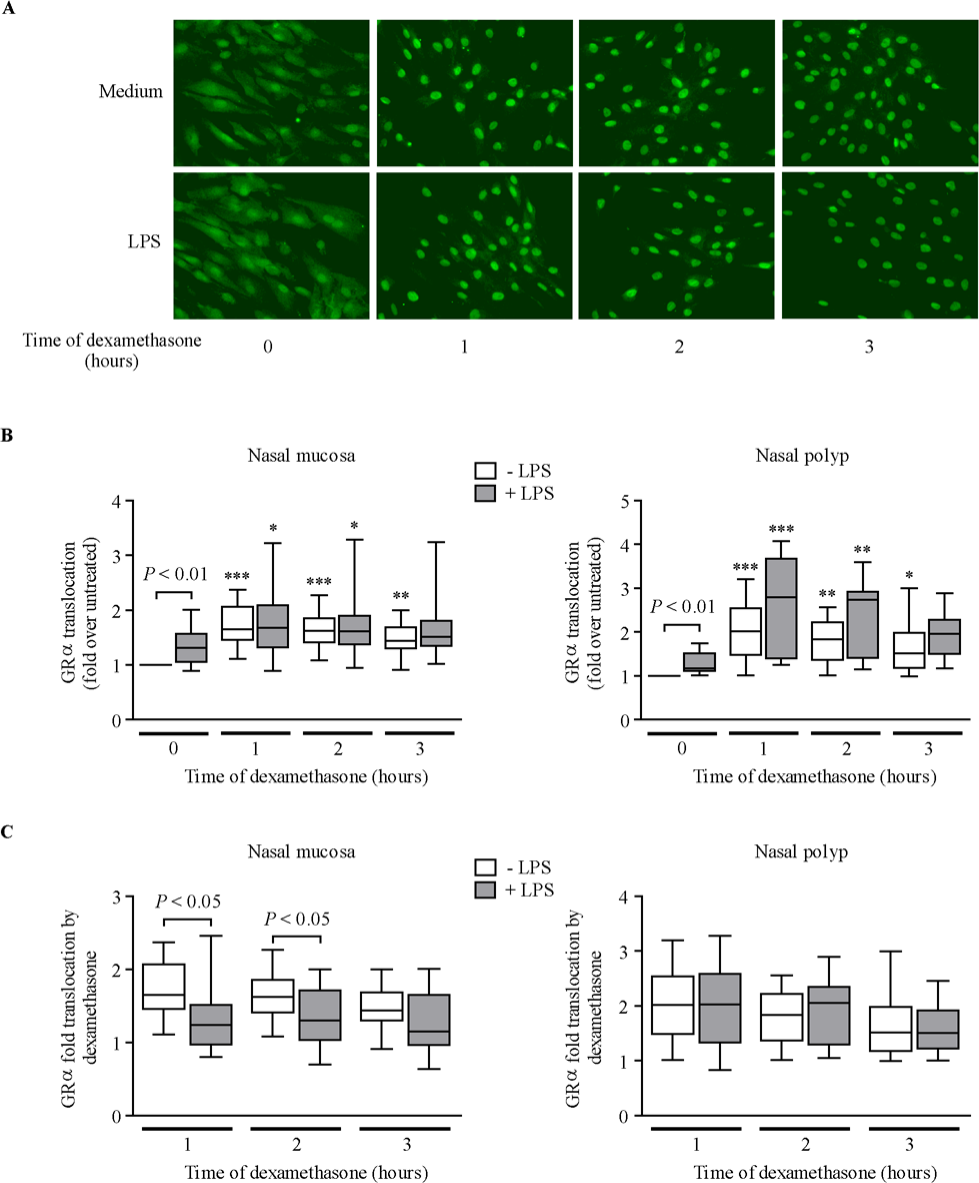
Effect of LPS on dexamethasone induction of GRα nuclear translocation. (A) GR immunofluorescence images of nasal fibroblasts pre-incubated with 10% csFBS-supplemented medium with/without LPS (10 μg/ml, 24 hours) prior to dexamethasone addition (10^–7^ M). (B) Quantification of GRα nuclear translocation in NM and NP fibroblasts (n = 12 each). *P<.05, **P<.01, and ***P<.001 *versus* 0 hours. (C) Ratio of GRα nuclear translocation induced by dexamethasone over time to the respective (medium or LPS) baseline (0 hours) values.

At baseline, GRα was homogenously distributed within the cell in medium-treated cells (Fig 4A). GRα nuclear immunoreactivity was higher in LPS-pre-incubated cells than in medium-treated cells (Fig 4B, 0 hours). Dexamethasone induced GRα translocation to the cell nucleus irrespective of LPS pre-incubation in both NM and NP fibroblasts (Fig 4B). However, after dividing the values of GRα nuclear translocation induced by dexamethasone over time by the respective (medium or LPS) baseline values, LPS reduced dexamethasone’s capacity to induce GRα translocation in NM fibroblasts (Fig 4C). GRβ nuclear levels remained invariable after dexamethasone and/or LPS cell stimulation (S3 Fig).

### Effect of LPS on GR transactivation of MKP-1 and GILZ

**Fig 5:**
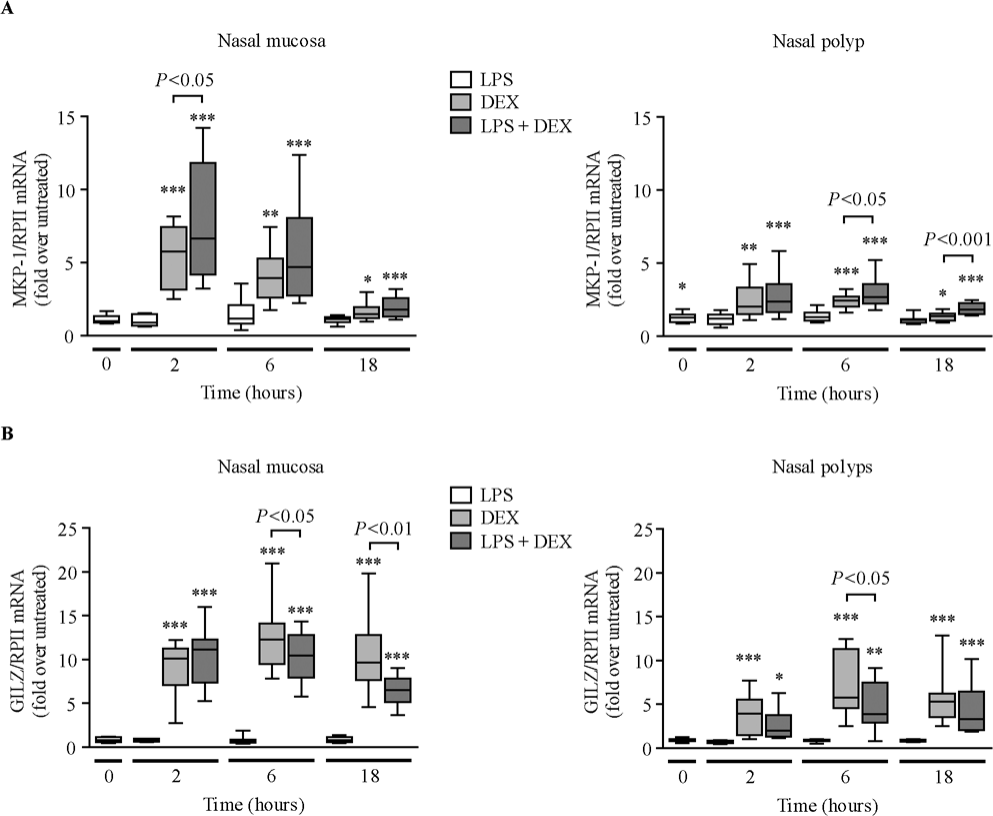
Effect of LPS on dexamethasone induction of *MKP-1* and *GILZ* gene expression. RT-PCR quantification of *MKP-1* (A) and *GILZ* (B) mRNAs in NM (n = 9–10) and NP (n = 12) fibroblasts pre-incubated with 10% csFBS-supplemented medium with/without LPS (10 μg/ml, 24 hours) prior to dexamethasone (DEX, 10^–7^ M) addition for the indicated times. *P<.05, **P<.01, and ***P<.001 *versus* untreated cells at each time point.

LPS pre-incubation did not significantly change *MKP-1* mRNA expression in NM fibroblasts, but increased *MKP-1* mRNA levels in NP fibroblasts (Fig 5A, 0 hours). LPS did not alter *GILZ* mRNA levels (Fig 5B). Dexamethasone induced the expression of *MKP-1* (Fig 5A) and *GILZ* mRNAs (Fig 5B). Dexamethasone induction of *MKP-1* mRNA was lower in NP than in NM fibroblasts [2 hours: 2.01 (median)-fold *vs* 5.76-fold, *P*<.001; 6 hours: 2.44-fold *vs* 3.93, *P*<.01]. Dexamethasone induction of *GILZ* mRNA was also lower in NP than in NM fibroblasts [2 hours: 3.93 (median)-fold *vs* 10.13-fold, *P*<.001; 6 hours: 5.77-fold *vs* 12.30, *P*<.01; 18 hours: 5.31-fold *vs* 9.65, *P*<.01]. LPS increased dexamethasone induction of *MKP-1* mRNA, but reduced dexamethasone induction of *GILZ* mRNA. However, after dividing the values of *MKP-1* mRNA induction by dexamethasone by the respective control (medium or LPS) values at each time point, LPS did not significantly reduce dexamethasone’s capacity to induce *MKP-1* and *GILZ* mRNA in nasal fibroblasts (S4 Fig).

**Fig 6:**
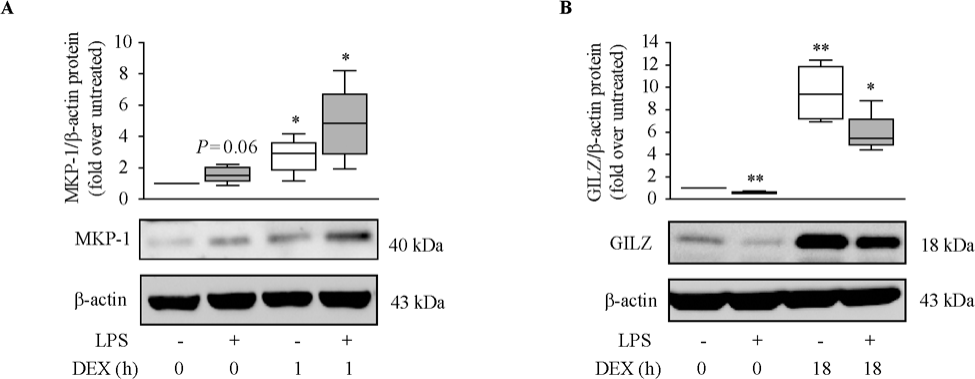
Effect of LPS on dexamethasone induction of MKP-1 and GILZ protein expression. Quantification of MKP-1 (**A**) and GILZ (**B**) proteins in NM fibroblasts (n = 4–5) pre-incubated with 10% FBS (**A**) or 10% csFBS-supplemented medium (**B**) with/without LPS (10 μg/ml, 24 hours) prior to dexamethasone (DEX, 10^–7^ M) addition for the indicated times. *P<.05 and **P<.01 *versus* untreated cells (0 hours).

The effect of LPS on dexamethasone-induced transactivation of MKP-1 and GILZ at the protein level was only analyzed in NM fibroblasts because these cells are the most sensitive to dexamethasone. LPS pre-incubation did not significantly change MKP-1 protein (Fig 6A) but decreased GILZ protein levels (Fig 6B). Dexamethasone induced the expression of MKP-1 and GILZ proteins. LPS pre-incubation increased dexamethasone induction of MKP-1 but decreased dexamethasone induction of GILZ protein. However, after dividing the values of MKP-1 or GILZ protein induction by dexamethasone by the respective control (medium or LPS), LPS did not significantly reduce dexamethasone’s capacity to induce MKP-1 and GILZ proteins (S5 Fig).

### Role of MKP-1 and GILZ on dexamethasone inhibition of cytokine production

**Fig 7:**
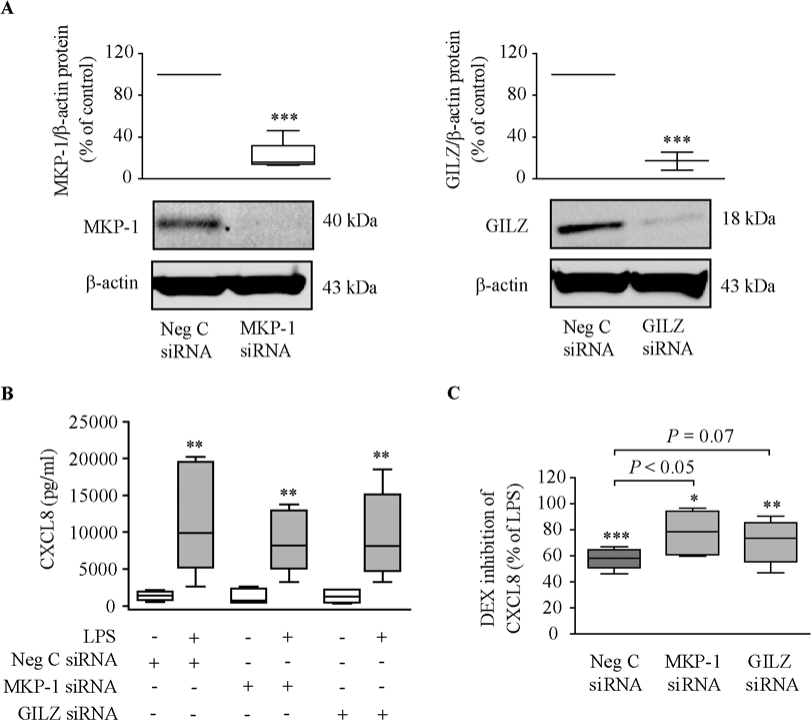
Role of MKP-1 and GILZ on dexamethasone inhibition of CXCL8 production. NM fibroblasts were transfected with MKP-1/GILZ/negative control siRNAs, as indicated in Methods. Twenty-four hours later, cells were pre-incubated with/without LPS (10 μg/ml, 24 hours) prior to incubation with 10% FBS-supplemented medium with/without dexamethasone (DEX, 10^–6^ M) for one (MKP-1 protein), six (GILZ protein) or twenty-four (CXCL8 release) hours. (**A**) MKP-1 and GILZ protein analysis (n = 3–4). ***P<.001 *versus* negative control siRNA. (**B**) FBS-induced CXCL8 production (n = 6). **P<.01 *versus* no LPS. (**C**) Dexamethasone inhibition of FBS-induced CXCL8 in LPS-pre-incubated cells (n = 6). *P<.05, **P<.01, and ***P<.001 *versus* LPS alone.

We examined the involvement of MKP-1 and GILZ on the inhibition of cytokine release by dexamethasone in six dexamethasone-sensitive NM fibroblast lines. Both MKP-1 and GILZ proteins were effectively knocked down in MKP-1 siRNA- and GILZ siRNA-transfected NM fibroblasts (Fig 7A). The induction of CXCL8 (Fig 7B) and IL-6 release (S6A Fig) by 10% FBS in LPS-pre-incubated cells did not differ between negative control siRNA-transfected cells and neither MKP-1 siRNA- nor GILZ siRNA-transfected cells. However, knockdown of MKP-1 reduced the capacity of dexamethasone to suppress FBS-induced CXCL8 release in LPS-pre-incubated cells (Fig 7C). This effect was not statistically significant (*P* =.07) for GILZ. Knockdown of MKP-1 and GILZ did not reduce dexamethasone’s capacity to inhibit the release of IL-6 (S6B Fig) and GM-CSF (S6C Fig) induced by FBS.

## Discussion

We report the *in vitro* effects of LPS on GR function in primary fibroblasts from control NM and in fibroblasts from patients with persistent chronic inflammation of the upper airways, *i*.*e*., patients with CRSwNP and asthma.

Previous studies have reported that nasal fibroblasts, as opposed to other airway fibroblasts, are particularly sensitive to LPS stimulation, in terms of induction of proinflammatory mediator release [22–24]. In our study, LPS pre-incubation enhanced the release of IL-6, CXCL8, RANTES, and GM-CSF induced by 10% FBS in both NM and NP fibroblasts. The enhancement of FBS-induced CXCL8 secretion in LPS-pre-incubated cells was higher in NP than in NM fibroblasts. Because these mediators actively participate in the recruitment and activation of inflammatory cells, their increased production in LPS-pre-treated nasal fibroblasts suggests that these cells probably contribute to perpetuate the local inflammatory response during bacterial-triggered exacerbations of the upper airways. In fact, it is known that LPS mimics the effects of Gram-negative bacteria. Thus, LPS and Gram-negative bacteria provoke a similar induction of CXCL8 in both airway smooth muscle cells [30] and BAL macrophages [13], and the Gram-negative bacterium *E*. *coli* induces GM-CSF release—but not eotaxin release—in airway smooth muscle cells, as occurs in LPS-treated nasal fibroblasts.

LPS effects in structural cells are known to occur via activation of toll-like receptors and also involving the activation of several intracellular signaling pathways, including MAPKs and NF-κB [31]. Our experiments using intracellular pathway-specific inhibitors revealed that activation of p38 MAPK and NF-κB is involved in the induction of IL-6 production by LPS in nasal fibroblasts. Interestingly, CXCL8 production by LPS was inhibited by p38 and JNK MAPK inhibitors and IKK/NF-κB inhibitor in NM but not in NP fibroblasts. This finding may be associated with the increased CXCL8 production in NP fibroblasts. These data suggest that these kinase pathways are over-activated in NP fibroblasts, inducing CXCL8 overproduction and possibly contributing to NP development.

The ability of dexamethasone to inhibit FBS-induced CXCL8, IL-6, and RANTES secretion was reduced in NM fibroblasts pre-incubated with LPS. In line with this, prolonged exposure of normal human monocytes to LPS provokes persistently increased IL-6 levels in the presence of dexamethasone [32]. Exposure of ovalbumin-sensitised mice to LPS makes allergic airway inflammation and hyper-responsiveness less responsive to dexamethasone [33]. In fact, infection by pathogenic microorganisms decreases glucocorticoid responsiveness in inflammatory airway diseases [11–13,19,34,35]. For instance, the capacity of dexamethasone to inhibit IL-6 and/or CXCL8 is reduced in primary human bronchial or transformed respiratory epithelial cells infected with either respiratory syncytial virus [19] or rhinovirus [11].

The lower capacity of dexamethasone to inhibit FBS-induced cytokine production in LPS-pre-incubated NM fibroblasts was not due to altered GRα/GRβ expression, but was associated with a modestly lower capacity of dexamethasone to induce GRα nuclear translocation. Infection of A549 cells with the respiratory syncytial virus does not alter GRα protein levels or GRα nuclear translocation [19], but infection of the same cells with rhinovirus impairs GRα nuclear translocation [11]. A number of other factors modulate the GR response, including the interaction with cofactors [36] and post-translational modifications of the GR, such as phosphorylation induced by different stimuli [37,38]. Rhinovirus infection of A549 cells increases GR Ser226 phosphorylation [11], which is known to blunt GR function. However, LPS pre-incubation does not appear to change GR Ser226 phosphorylation status in our nasal fibroblasts (unpublished data).

In immune cells, such as macrophages, MKP-1 expression is up-regulated in response to a wide variety of proinflammatory stimuli, including ligands of the Toll-like receptors [17]. This increase is a regulatory mechanism that inhibits MAPK activation and helps to terminate the inflammatory cascade. However, LPS pre-incubation did not significantly increase MKP-1 expression in nasal fibroblasts. In contrast, LPS decreased GILZ protein expression in nasal fibroblasts. This finding concurs with the reported GILZ down-regulation after stimulation of human alveolar macrophages with LPS [39]. Noteworthy, LPS did not reduce the capacity of dexamethasone to induce MKP-1 and GILZ expression in nasal fibroblasts. This finding suggests that LPS does not affect the capacity of glucocorticoids to transactivate anti-inflammatory genes in these cells. This contrasts with the attenuation of dexamethasone’s ability to induce the expression of *MKP-1* mRNA in A549 cells and in primary bronchial epithelial cells infected with rhinovirus [11], and in BAL macrophages incubated with either LPS or the bacterial pathogen *H*. *parainfluenzae* [13]. These discrepancies may be due to differences in the stimulus/agent used and/or the cell type used.

We finally sought to determine whether transactivation of MKP-1 and GILZ by glucocorticoids plays an anti-inflammatory role in nasal fibroblasts. MKP-1 deficiency in macrophages results in excessive production of numerous proinflammatory cytokines [17]. In contrast, silencing of MKP-1 in nasal fibroblasts did not provoke an increase of FBS-induced CXCL8 and IL-6 production. However, silencing of MKP-1 reduced the capacity of dexamethasone to inhibit FBS-induced CXCL8 production. This suggests that inhibition of CXCL8 by glucocorticoids occurs, at least in part, through glucocorticoid-induced MKP-1 transactivation. These results are in line with those performed in macrophages from MKP-1 knockout mice and in cells with silenced expression of MKP-1 [40]. Noteworthy, the lower capacity of dexamethasone to decrease CXCL8 production in NP fibroblasts compared to NM fibroblasts may be due to the lower induction of MKP-1 expression by glucocorticoids in NP fibroblasts. MKP-1 silencing did not alter the inhibition of IL-6 and GM-CSF by dexamethasone. This suggests that the inhibition of these cytokines by glucocorticoids does not occur through glucocorticoid-induced MKP-1 transactivation. Our findings are consistent with the concept that not all anti-inflammatory effects of glucocorticoids are mediated through MKP-1 [17].

In line with studies in circulating monocytes [41], silencing of GILZ in nasal fibroblasts did not increase FBS-induced CXCL8 and IL-6 production. GILZ silencing in nasal fibroblasts mildly, but not significantly, attenuated dexamethasone’s inhibition of FBS-induced CXCL8 production. GILZ silencing did not affect the inhibition of IL-6 and GM-CSF by dexamethasone. GILZ knockdown in HEK293 cells markedly inhibited the ability of dexamethasone to suppress IL-1β-induced CXCL8 production [42], and GILZ knockdown in circulating monocytes reduced the inhibitory effect of glucocorticoids on TNF-α and RANTES secretion [41]. Overall, the involvement of MKP-1 and GILZ in glucocorticoid anti-inflammatory effects depends on the cell type and the proinflammatory mediator analyzed.

In summary, pre-incubation of nasal fibroblasts with LPS enhances the secretion of the proinflammatory mediators IL-6, CXCL8, RANTES, and GM-CSF induced by 10% FBS. FBS-induced CXCL8 secretion is higher in NP than in NM fibroblasts. LPS pre-incubation reduces the capacity of dexamethasone to inhibit FBS-induced IL-6, CXCL8 and RANTES production, decreases dexamethasone-induced GRα nuclear translocation (only in NM fibroblasts), does not alter GRα/GRβ expression, decreases GILZ expression, but does not decrease the capacity of dexamethasone to induce GILZ and MKP-1 expression. Finally, we show that glucocorticoid-induced transactivation of MKP-1 is involved in CXCL8 inhibition by dexamethasone. In conclusion, the bacterial product LPS negatively affects GR function in control NM and NP fibroblasts by interfering with the capacity of the activated receptor to inhibit the production of pro-inflammatory mediators. This study contributes to the understanding of how bacterial infection of the upper airways may limit the efficacy of glucocorticoid treatment.

## Supporting Information

S1 Dataset Individual data points (raw data) of Figs 1, 2, 3, 4, 5, 6 and 7 and S1, S2, S3, S4, S5 and S6 Figs. (XLS)

S1 Fig Effect of LPS on dexamethasone inhibition of IL-6 secretion. ELISA quantification of IL-6 production in cell supernatants of NM and NP fibroblasts (n = 8 each) pre-incubated with 10% csFBS-supplemented medium with/without LPS (10 μg/ml, 24 hours) and then incubated with 10% FBS-supplemented medium with/without dexamethasone (DEX) for 24 hours. IL-6 production normalized to each respective control. *P<.05 and ***P<.001 *versus* medium (no DEX).
(TIF)

S2 Fig Effect of LPS on GR expression. Quantification of total GRα and nuclear GRβ proteins in NM and NP fibroblasts (n = 5–7) pre-incubated with 10% csFBS-supplemented medium with/without LPS (10 μg/ml) for 24 hours.
(TIF)

S3 Fig Effect of LPS on GRβ nuclear translocation. Quantification of GRβ nuclear translocation in response to dexamethasone (10^–7^ M, 1 hour) in NM and NP (n = 5 each) fibroblasts pre-incubated with 10% csFBS-supplemented medium with/without LPS for 24 hours. Representative GRβ Western blot images are shown. Lamin B was used as loading control and α-tubulin was used to assure purity of the nuclear extracts.
(TIF)

S4 Fig Effect of LPS on dexamethasone induction of *MKP-1* and *GILZ* gene expression. RT-PCR quantification of *MKP-1* (**A**) and *GILZ* (**B**) mRNAs in NM (n = 9–10) and NP (n = 12) fibroblasts pre-incubated with 10% csFBS-supplemented medium with/without LPS (10 μg/ml, 24 hours) prior to dexamethasone (DEX, 10^–7^ M) addition for the indicated times. Data show the ratio of *MKP-1* or *GILZ* mRNA induction by dexamethasone to the respective control (medium or LPS) at each time point.
(TIF)

S5 Fig Effect of LPS on dexamethasone induction of MKP-1 and GILZ protein expression. Quantification of MKP-1 (**A**) and GILZ (**B**) proteins in NM fibroblasts (n = 4–5) pre-incubated with 10% FBS (**A**) or 10% csFBS-supplemented medium (**B**) with/without LPS (10 μg/ml, 24 hours) prior to dexamethasone (DEX, 10^–7^ M) addition for one (**A**) or 18 hours (**B**). Data show the ratio of MKP-1 or GILZ protein induction by dexamethasone to the respective control (medium or LPS).
(TIF)

S6 Fig Role of MKP-1 and GILZ on dexamethasone inhibition of IL-6 and GM-CSF production. NM fibroblasts (n = 6) were transfected with MKP-1/GILZ/negative control siRNAs, as indicated in Methods. Twenty-four hours later, cells were pre-incubated with/without LPS (10 μg/ml, 24 hours) prior to incubation with 10% FBS-supplemented medium with/without dexamethasone (DEX, 10^–6^ M) for 24 hours. (**A**) FBS-induced IL-6 production. **P<.01 *versus* no LPS. Dexamethasone inhibition of FBS-induced IL-6 (**B**) and GM-CSF (**C**) release in LPS-pre-incubated cells. ***P<.001 *versus* LPS alone.
(TIF)

S7 Fig Original uncropped Western blot images of Fig 6A and 6B. (TIF)

S8 Fig Original uncropped Western blot images of Fig 7A. (TIF)

S9 Fig Original uncropped Western blot images of S3 Fig. (TIF)

S1 Table Epidemiological characteristics of the study population. (DOC)
