# Supplementary figures and images for: Effect of Lipopolysaccharide on Glucocorticoid Receptor Function in Control Nasal Mucosa Fibroblasts and in Fibroblasts from Patients with Chronic Rhinosinusitis with Nasal Polyps and Asthma
Source: PLoS One. 2015 May 5;10(5):e0125443. doi: 10.1371/journal.pone.0125443 (PMC4420770; doi:10.1371/journal.pone.0125443)

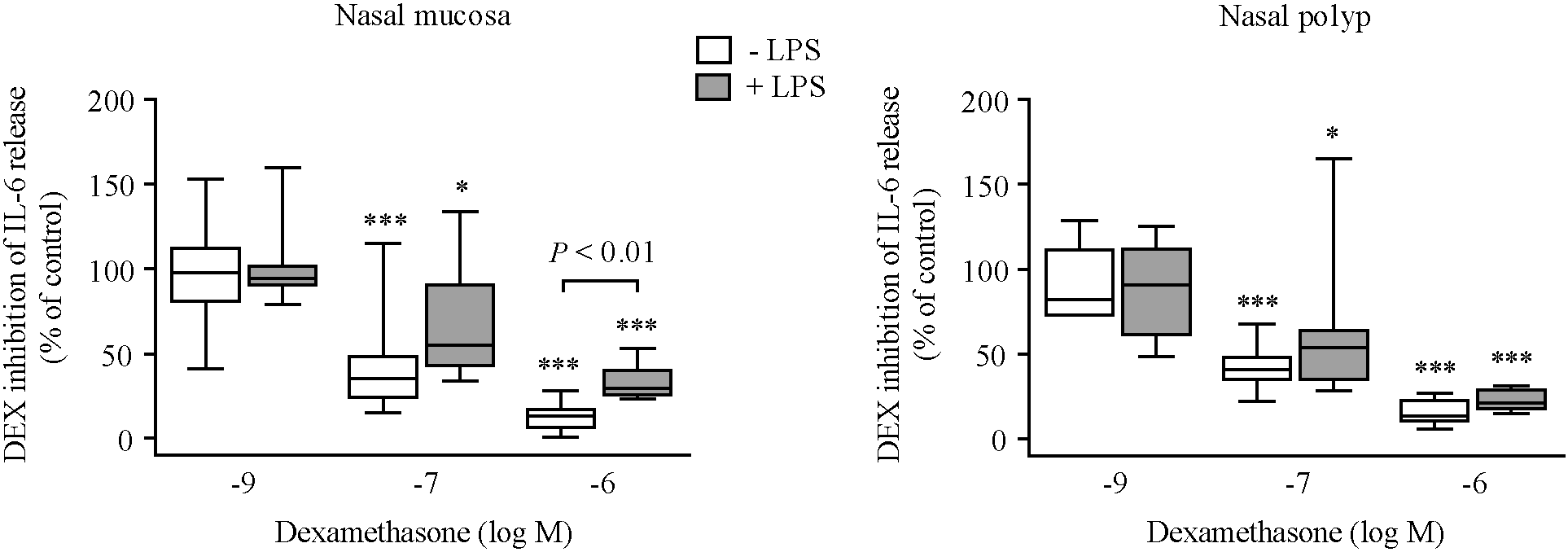

Supplement: S1 Fig — ELISA quantification of IL-6 production in cell supernatants of NM and NP fibroblasts (n = 8 each) pre-incubated with 10% csFBS-supplemented medium with/without LPS (10 μg/ml, 24 hours) and then incubated with 10% FBS-supplemented medium with/without dexamethasone (DEX) for 24 hours. IL-6 production normalized to each respective control. *P<.05 and ***P<.001 versus medium (no DEX). (TIF) [file pone.0125443.s002.tif]

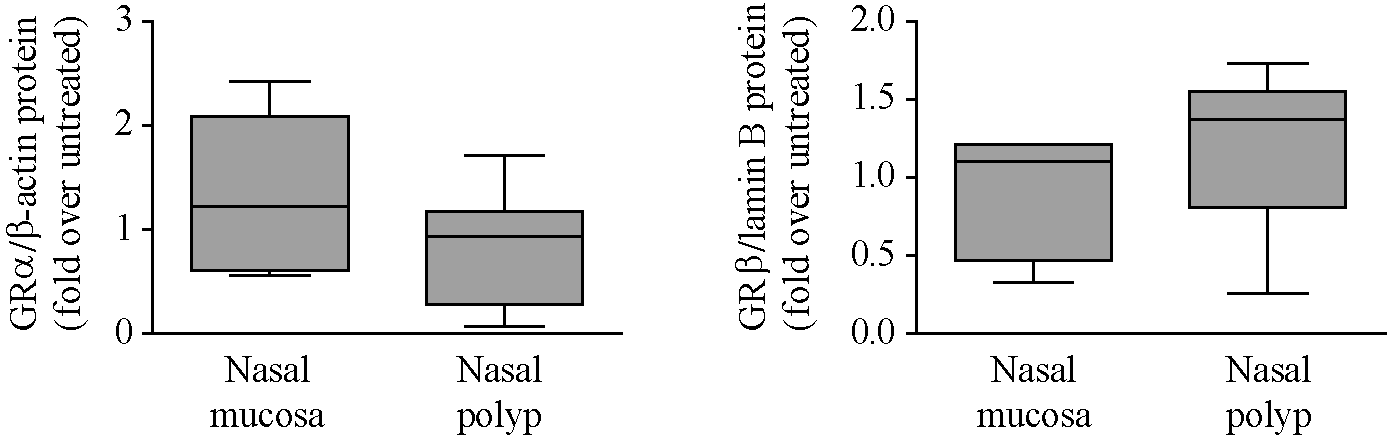

Supplement: S2 Fig — Quantification of total GRα and nuclear GRβ proteins in NM and NP fibroblasts (n = 5–7) pre-incubated with 10% csFBS-supplemented medium with/without LPS (10 μg/ml) for 24 hours. (TIF) [file pone.0125443.s003.tif]

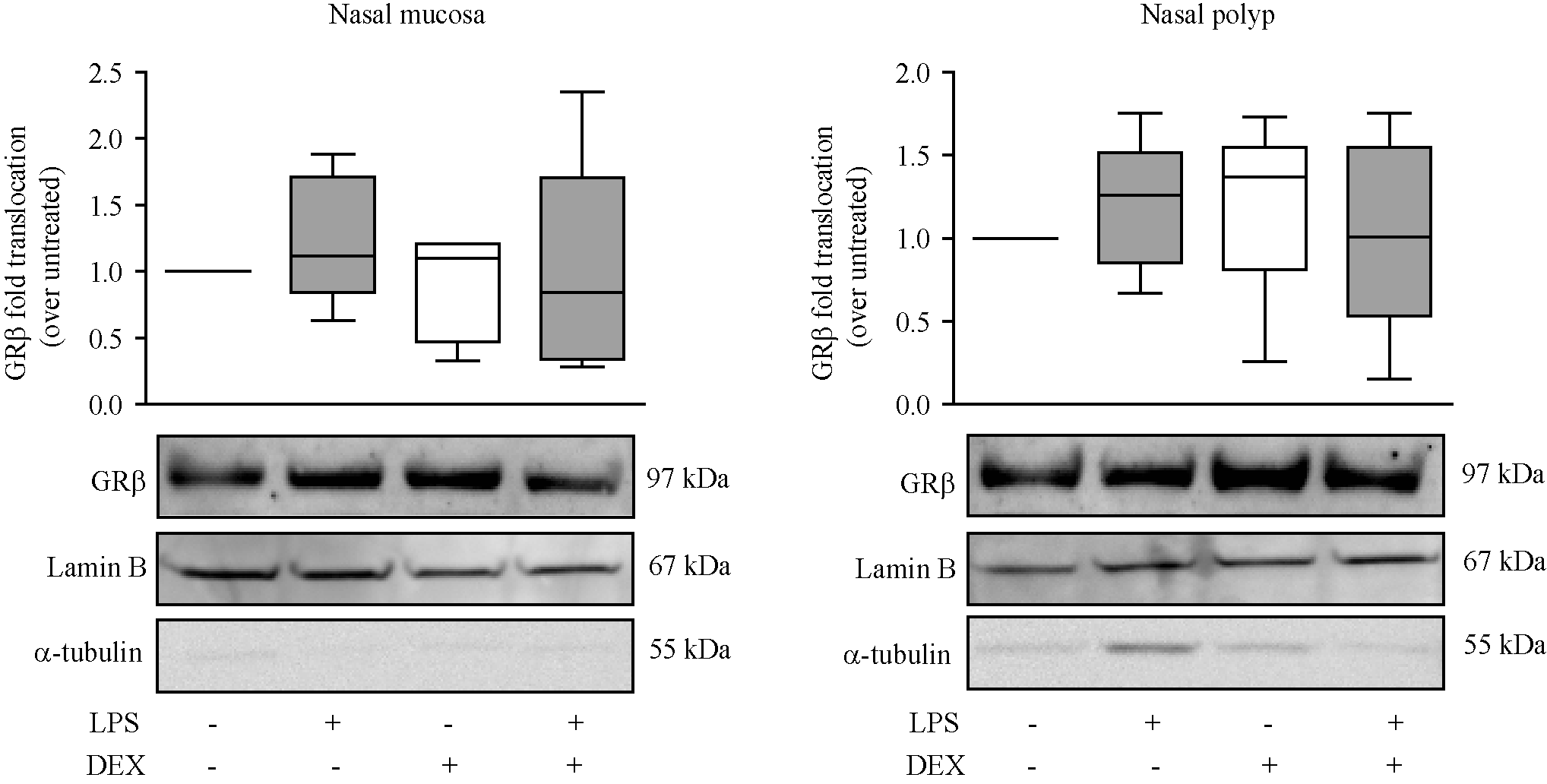

Supplement: S3 Fig — Quantification of GRβ nuclear translocation in response to dexamethasone (10–7 M, 1 hour) in NM and NP (n = 5 each) fibroblasts pre-incubated with 10% csFBS-supplemented medium with/without LPS for 24 hours. Representative GRβ Western blot images are shown. Lamin B was used as loading control and α-tubulin was used to assure purity of the nuclear extracts. (TIF) [file pone.0125443.s004.tif]

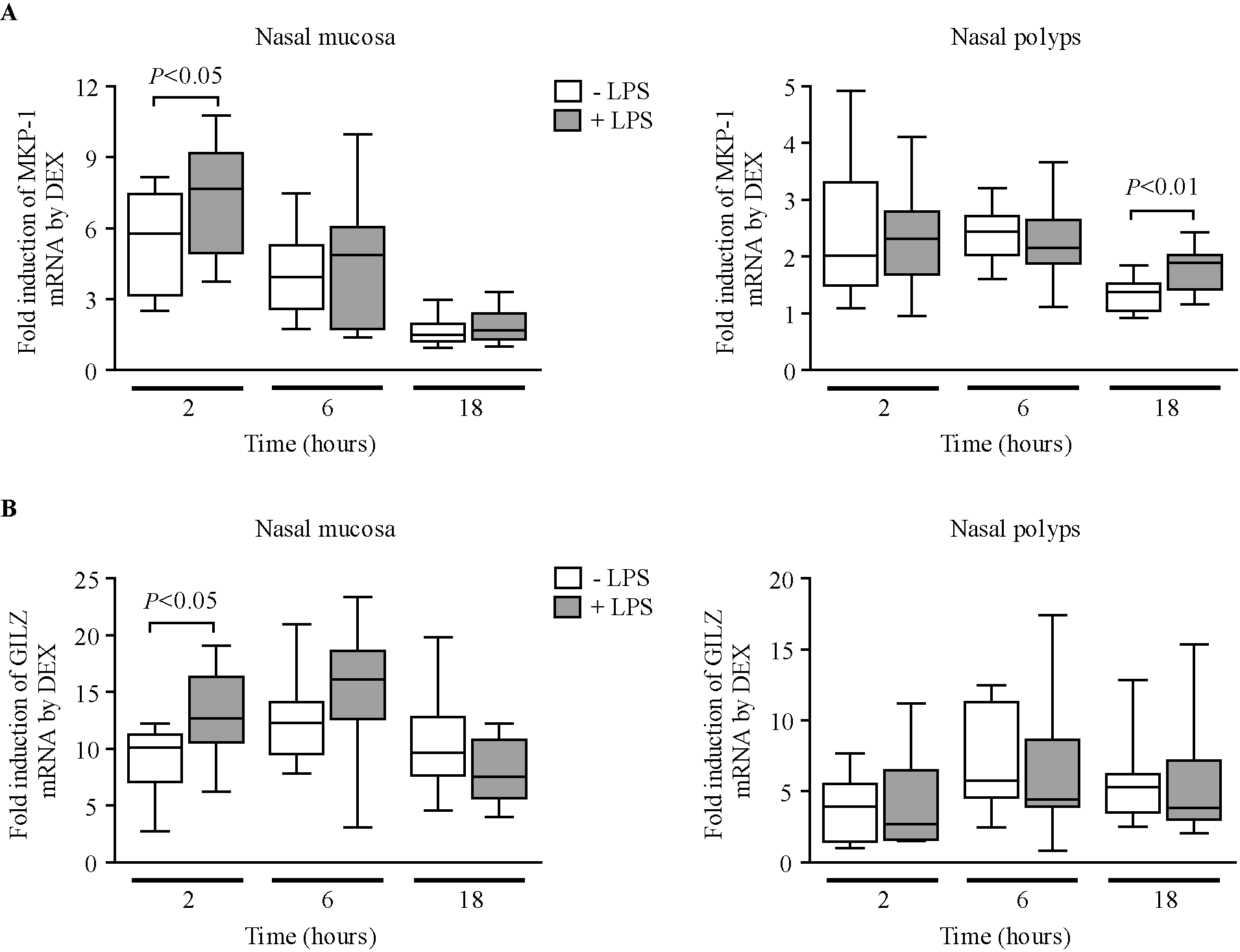

Supplement: S4 Fig — RT-PCR quantification of MKP-1 (A) and GILZ (B) mRNAs in NM (n = 9–10) and NP (n = 12) fibroblasts pre-incubated with 10% csFBS-supplemented medium with/without LPS (10 μg/ml, 24 hours) prior to dexamethasone (DEX, 10–7 M) addition for the indicated times. Data show the ratio of MKP-1 or GILZ mRNA induction by dexamethasone to the respective control (medium or LPS) at each time point. (TIF) [file pone.0125443.s005.tif]

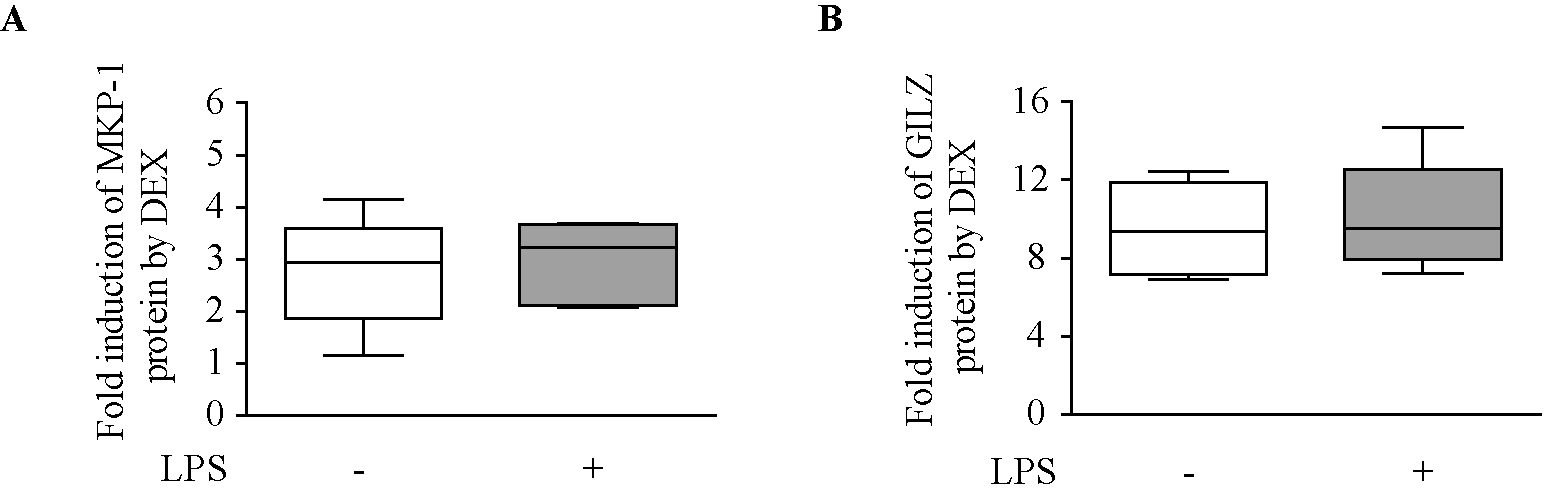

Supplement: S5 Fig — Quantification of MKP-1 (A) and GILZ (B) proteins in NM fibroblasts (n = 4–5) pre-incubated with 10% FBS (A) or 10% csFBS-supplemented medium (B) with/without LPS (10 μg/ml, 24 hours) prior to dexamethasone (DEX, 10–7 M) addition for one (A) or 18 hours (B). Data show the ratio of MKP-1 or GILZ protein induction by dexamethasone to the respective control (medium or LPS). (TIF) [file pone.0125443.s006.tif]

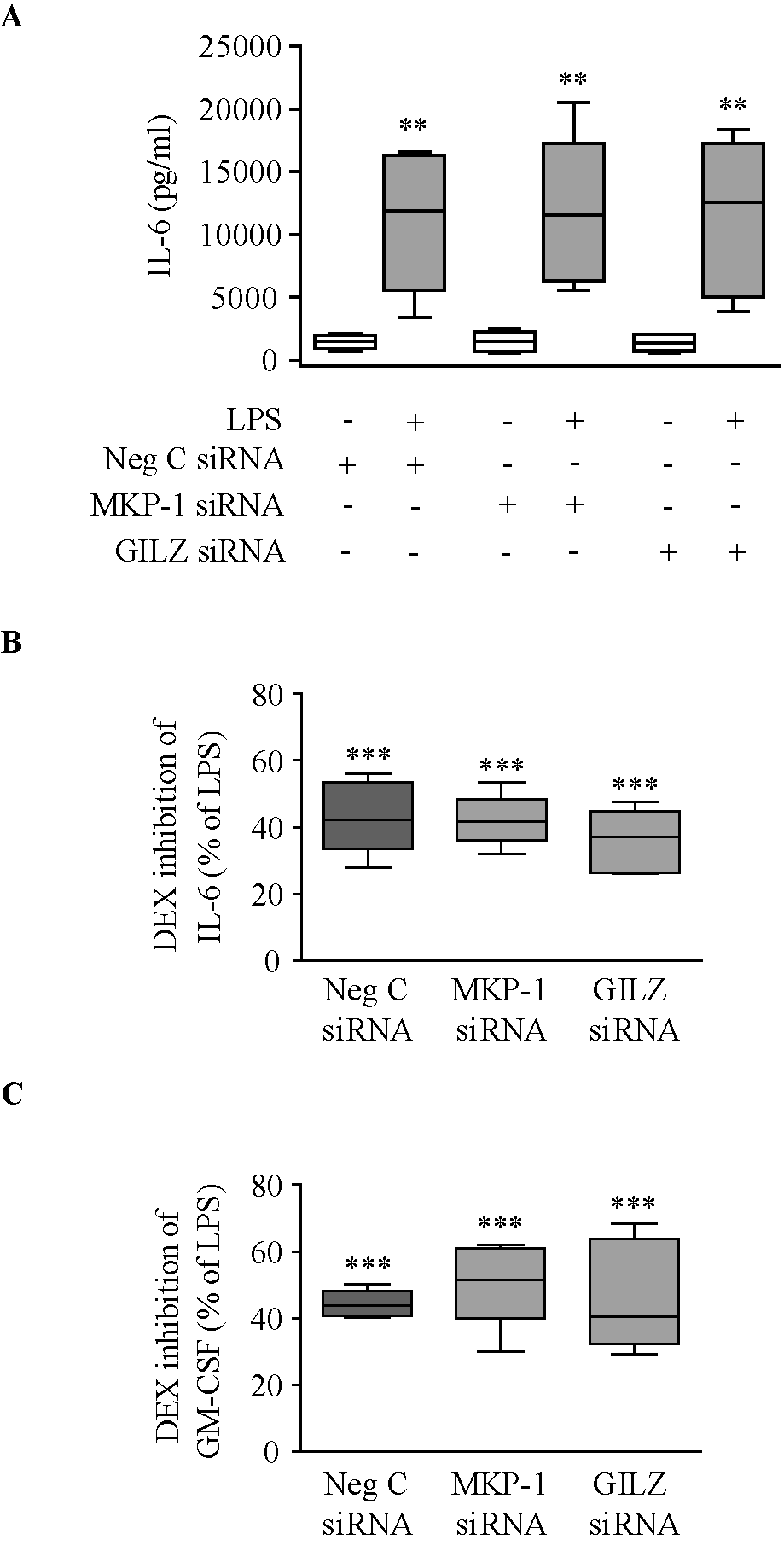

Supplement: S6 Fig — NM fibroblasts (n = 6) were transfected with MKP-1/GILZ/negative control siRNAs, as indicated in Methods. Twenty-four hours later, cells were pre-incubated with/without LPS (10 μg/ml, 24 hours) prior to incubation with 10% FBS-supplemented medium with/without dexamethasone (DEX, 10–6 M) for 24 hours. (A) FBS-induced IL-6 production. **P<.01 versus no LPS. Dexamethasone inhibition of FBS-induced IL-6 (B) and GM-CSF (C) release in LPS-pre-incubated cells. ***P<.001 versus LPS alone. (TIF) [file pone.0125443.s007.tif]

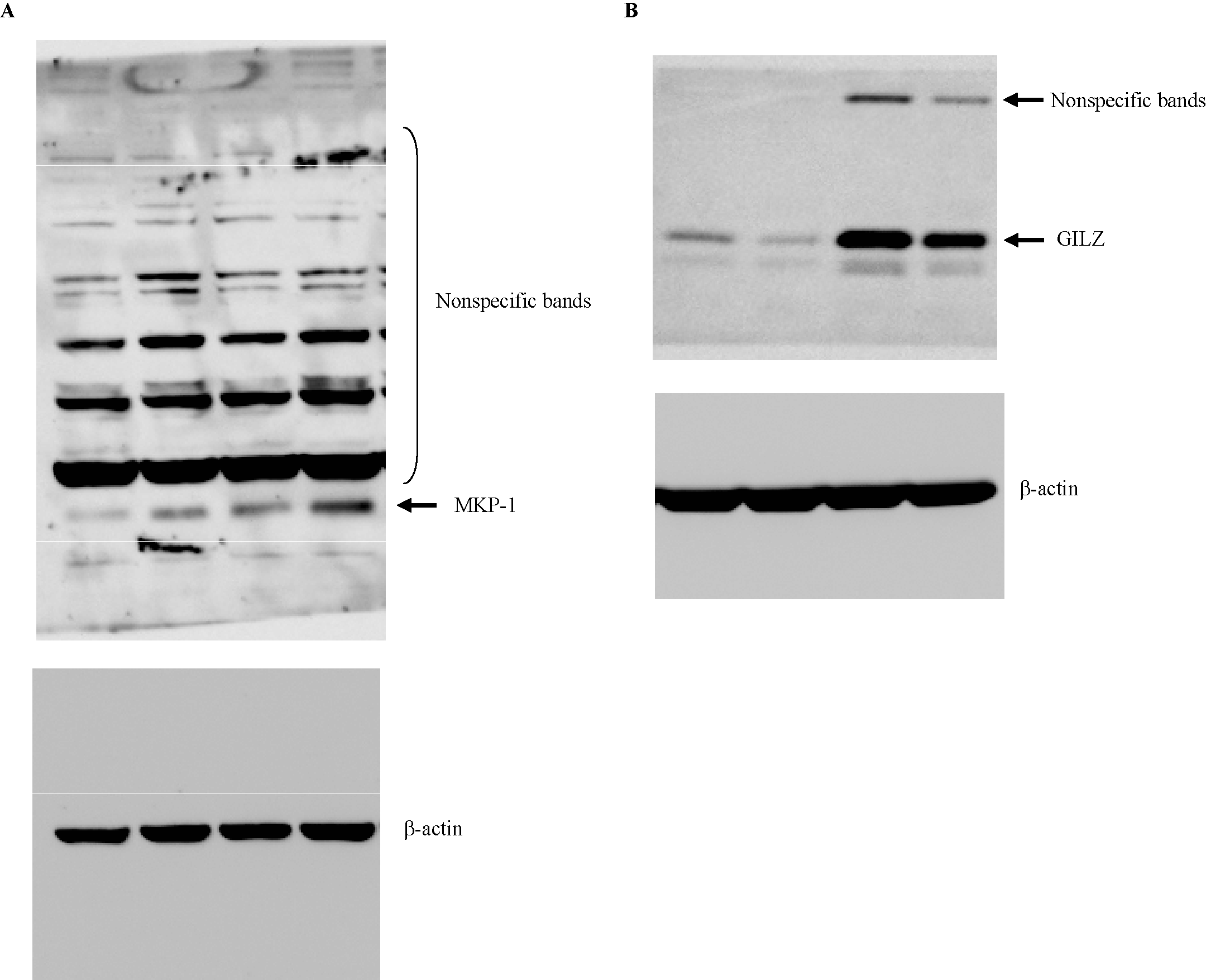

Supplement: S7 Fig — (TIF) [file pone.0125443.s008.tif]

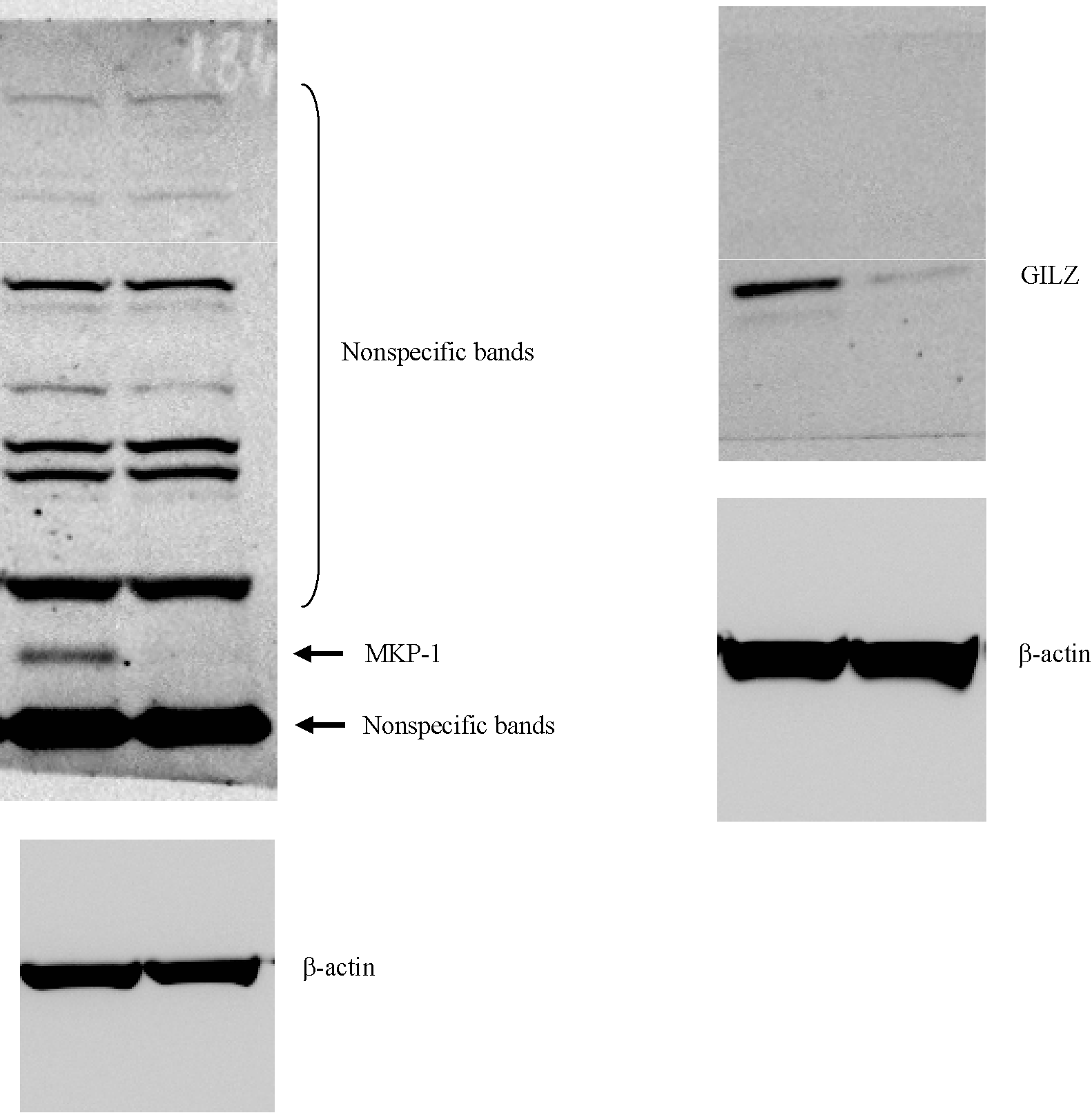

Supplement: S8 Fig — (TIF) [file pone.0125443.s009.tif]

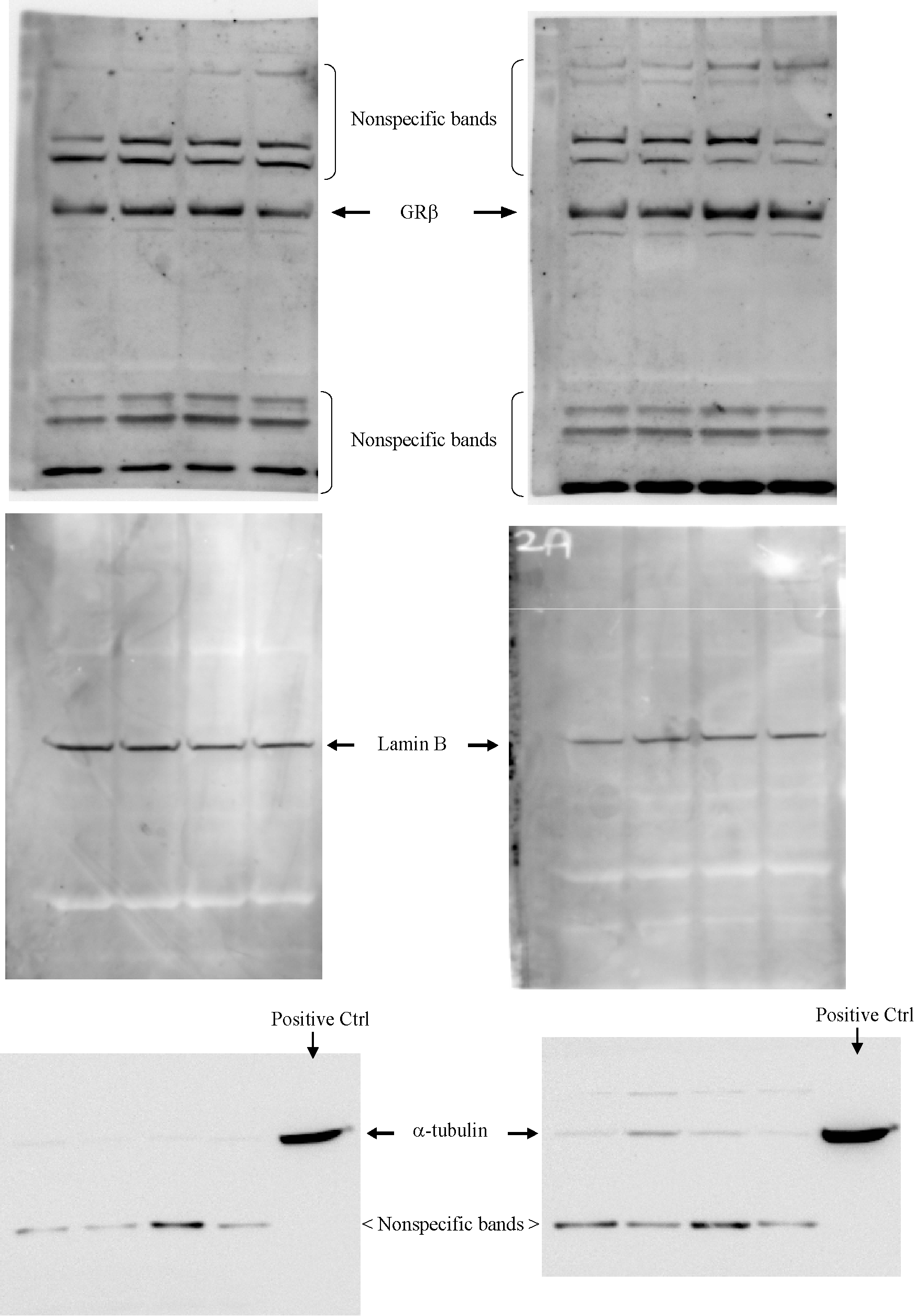

Supplement: S9 Fig — (TIF) [file pone.0125443.s010.tif]
