# Supplementary material for: Effect of Lipopolysaccharide on Glucocorticoid Receptor Function in Control Nasal Mucosa Fibroblasts and in Fibroblasts from Patients with Chronic Rhinosinusitis with Nasal Polyps and Asthma
Source: PLoS One. 2015 May 5;10(5):e0125443. doi: 10.1371/journal.pone.0125443 (PMC4420770; doi:10.1371/journal.pone.0125443)
Supplement: S1 Table — (DOC) [file pone.0125443.s011.doc]

**S1 Table.** Epidemiological characteristics of the study population.

| **Characteristics** | **Nasal mucosa** | **Nasal polyp** |
| --- | --- | --- |
| Subjects (n) | 10 | 12 |
| Age, years (mean ± SD) | 42.8 ± 15.7 | 46 ± 14.2 |
| Females, n (%) | 2 (20) | 6 (50) |
| Asthma, n (%) | 0 (0) | 12 (100) |
| Aspirin-exacerbated respiratory disease, n (%) | 0 (0) | 7 (58.3) |
| Intranasal glucocorticoids, n (%) | 0 | 12 (100) |
| Skin prick test positivity, n (%) | 1 (10) | 3 (25) |
